# Isolation and genetic characterization of waterfowl parvovirus in ducks in Northern Vietnam

**DOI:** 10.14202/vetworld.2024.981-987

**Published:** 2024-05-04

**Authors:** Nguyen Thi Huong, Dong Van Hieu, Nguyen Thi Bich, Tran Van Khanh, Nguyen Thanh Ba, Chu Thi Ngoc Xuan, Quach Thi Minh Hien, Truong Ha Thai, Chu Thi Thanh Huong

**Affiliations:** 1 Hanvet Pharmaceutical Company Limited, Hanoi, Vietnam; 2 Department of Veterinary Public Health, Faculty of Veterinary Medicine, Vietnam National University of Agriculture, 12400, Hanoi, Vietnam; 3 Department of Microbiolgy – Infectious disease, Faculty of Veterinary Medicine, Vietnam National University of Agriculture, 12400, Hanoi, Vietnam

**Keywords:** Goose parvovirus, isolation, phylogenetic analysis, Vietnam, virulence

## Abstract

**Background and Aim:**

Short beak and dwarfism syndrome (SBDS), a highly contagious disease, has been reported in duck farms in Vietnam since 2019. In this study, we evaluated the virulence and characterized the virus obtained from SBDS cases in North Vietnam.

**Materials and Methods:**

Polymerase chain reaction was used to detect waterfowl parvovirus in ducks, and the virus from positive samples was inoculated into 10-day-old duck-embryonated eggs to reproduce the disease in young ducklings to determine the virulence and subjected to phylogenetic analysis of non-structural (NS) and VP1 gene sequences.

**Results and Discussion:**

Goose parvovirus (GPV) was isolated from ducks associated with SDBS in Vietnam. The virus Han-GPV2001 is highly virulent when inoculated into 10-day-old duck embryos and 3-day-old ducklings. The mortality rate of duck embryos was 94.35% within 6 days of virus inoculation. Inoculating 3-day-old ducks with the virus stock with 10^4.03^ EID_50_ through intramuscular and neck intravenous administration resulted in 80% and 66.67% of clinical signs of SDBS, respectively, were shown. Phylogenetic analysis based on the partial NS and VP1 gene sequences revealed that the viral isolate obtained in this study belonged to novel GPV (NGPV) and was closely related to previous Vietnamese and Chinese strains.

**Conclusion:**

A GPV strain, Han-GPV2001, has been successfully isolated and has virulence in duck-embryonated eggs as well as caused clinical signs of SBDS in ducks. Phylogenetic analyses of partial genes encoding NS and capsid proteins indicated that the obtained GPV isolate belongs to the NGPV group.

## Introduction

Ducking short beak and dwarfism syndrome (SBDS) is a highly contagious disease caused by goose parvovirus (GPV) in ducks. Affected ducks showed growth retardation, short beaks, tongue protrusions, and loss of appetite [1]. It was first reported in France in mule duck flocks during the 1970s [2] and subsequently emerged in Taiwan in 1989 [3], Poland in 1995 [2, 4], and China in 2014 [5]. In Vietnam, a virus associated with SBDS was reported in 2019 [6]. A water fowl-gene positive rate of 20% was observed among ducks raised in several cities and provinces in Northern Vietnam [7].

Waterfowl parvovirus belongs to the genus *Dependoparvovirus* of the family *Parvoviridae* and is a non-enveloped and single-stranded DNA virus. The viral genome consists of two open reading frames, the left encoding the non-structural (NS) protein Rep and the right encoding structural proteins VP1, VP2, and VP3 [8]. The VP1 gene sequence was variable among waterfowl parvoviruses, which shared 85% identity between GPV and Muscovy duck parvovirus (MDPV). The previous studies by in China, Taiwan [9, 10], and Vietnam [7] used VP1 and complete genome to characterize viral strains. Analysis of the complete genome of four waterfowl parvovirus strains revealed that the Vietnamese strains belonged to novel GPV (NGPV) and resulted from a recombination event [10].

In this study, viruses were isolated from ducks showing clinical signs of SBDS. The virulence of the virus was evaluated, and its characteristics were characterized in SBDS cases in North Vietnam.

## Materials and Methods

### Ethical approval

Samples were collected from duck farms in northern Vietnam under the auspices of the Vietnam National University of Agriculture and the protocol for sampling purposes was submitted and approved by the Committee on Animal Research and Ethics of the University (CARE-2020/14). Verbal consent was obtained from the duck farm owners before sampling.

### Study period and location

The study was conducted from 2020 to 2022 in laboratory of Hanvet pharmaceutical company limited, located at Pho Noi A Industrial Zone, My Hao Town, Hung Yen Province, Vietnam.

### Sampling

In total, 17 pooled tissue samples (consisting heart, liver, and spleen) were collected from ducks in Ha Noi, Bac Giang, Thai Binh, Hai Phong, Hung Yen, Ha Nam, Hai Duong, and Bac Ninh provinces in Northern Vietnam (Table-1). There was no vaccination with waterfowl parvovirus in these farms. These ducks, which showed typical symptoms of SBDS, were selected for sampling. Samples were kept on ice to the laboratory, then were homogenized in 10% phosphate-buffered saline and stored at - 80°C until use.

**Table 1:** Information of obtained samples using in this study.

No.	Name	Province/city	Days of age
1	Han - GPV1901	Bac Giang	19
2	Han - GPV1902	Bac Giang	30
3	Han - GPV1903	Hai Phong	20
4	Han - GPV1904	Thai Binh	18
5	Han - GPV1905	Bac Giang	60
6	Han - GPV1906	Ha Noi	21
7	Han - GPV1907	Thai Binh	78
8	Han - GPV1908	Hung Yen	15
9	Han - GPV1909	Hung Yen	22
10	Han - GPV1910	Ha Nam	15
11	Han - GPV1911	Hai Duong	20
12	Han- GPV1912	Hai Duong	45
13	Han - GPV2001	Bac Giang	14
14	Han - GPV2002	Bac Giang	21
15	Han - GPV2007	Hung Yen	21
16	Han - GPV2103	Thai Binh	21
17	Han - GPV2104	Bac Ninh	21

GPV=Goose parvovirus

### Virus isolation

Virus isolation was performed as previously described by Chen *et al*. [5], Wan *et al*. [11], and Shao *et al*. [12]. In brief, homogenized samples were freeze-thawed 3 times, centrifuged at 8000× *g* for 15 min at 4°C, and the supernatant was passed through a filter with a pore diameter of 0.22 μm. The presence of waterfowl parvovirus was detected by polymerase chain reaction (PCR) using the specific primers NSF1/NSR1 (Table-2) [13–19]. If the test results were negative, the sample was continuously inoculated for five passages and the presence of parvovirus was again detected by PCR.

**Table 2:** The primes used in this study.

Name	Nucleotide sequences (5’- 3’)	PCR products (bp)	Reference
NSF1	CAATGGGCTTTTACCAATATGC	641	[13]
NSR1	ATTTTTCCCTCCTCCCACCA		
P6F	CTACAACCCGGACCTGTGTC	921	[14]
P6R	GCATGCGCGTGGTCAACCTAACA		
TMuVE-F	GAAGCGAGCACCTACCACA	249	[15]
TMuVE-R	CGCTGATGACCCTGTCCAT		
DHV-3DF	ACAATGACCCAGCCTTAG	440	[16]
DHV-3DR	CCACTGTATCTTCCCTTC		
DHAV-1F	CAACTCGACCAATHCCTGG	492	
DHAV-1R	CCTGRTGRACCATTGTRACTG		
P1F	TTGATGGCAGGCCTCTTGC	354	[17]
P2R	GGAGGATGTTGGCAGCATT		
Flu A-F	CTTCTAACCGAGGTCGAAAC	244	[18]
Flu A- R	AGGGCATTTTGGACAAAKCGTCTA		
DEV-7F	GAAGGCGGGTATGTAATGTA	446	[19]
DEV-7R	CAAGGCTCTATTCGGTAATG		

PCR=Polymerase chain reaction

### Hemagglutination assay

Allantoic fluid samples collected from duck eggs were used for the hemagglutination assay according to the method described by Wang *et al*. [20].

### Determination of virus virulence

Methods for infecting and determining the virulence of the isolated virus strain were as described previously by Palya *et al*. [2] and Wan *et al*. [13]. The virus used in this study was obtained from five duck embryo passages. The duck weight was monitored on 0, 10, 17, 24, 29, 36, and 51 days after the injection. Clinical signs, such as short beak, shrunken legs, low body weight, and death, were observed daily. Viremia and viral clearance were detected on 0, 7, 14, 21, 28, and 35 days after infection. The weight and size of the beak were statistically analyzed using the analysis of variance function on Minitab software with 95% confidence.

### DNA extraction and PCR

Total DNA was extracted using the WizPrep™ Viral DNA/RNA Mini Kit (Wizbiosoluions, Gyeonggi-do, Korea). DNA was suspended in 50 μL of distilled water and stored at –30°C until use. PCR was performed using GoTaq® Green Master Mix (Promega, Madison, WI, USA) and specific primers for each virus to detect waterfowl parvovirus and avoid coinfection with some viruses in the duck genome (such as influenza A, duck enteritis virus, duck hepatitis A virus, Newcastle disease virus and Tembusu virus) (Table-2) [13–19]. PCR products were electrophoresed on 1.5% agarose gels and visualized under UV light.

### Nucleotide sequencing and analysis

PCR products were amplified using two pairs of primers (NSF1/NSR1 and P6F/P6R) and electrophoresed on a 1.5% agarose gel. Predicted products were extracted from the gel and purified using the GeneJET Gel Extraction Kit (Thermo Fisher Scientific Baltics UAB V.A. Graiciuno 8, LT-02241 Vilnius, Lithuania). Purified products were sent to Malaysian 1^st^ BASE for sequencing. Sequence data were aligned using BioEdit software (https://bioedit.software.informer.com/) [21]. We compared the partial NS and VP1 nucleotide sequences of the isolate obtained in this study with other sequences published in GenBank (https://www.ncbi.nlm.nih.gov/). Subsequently, the phylogenetic trees were constructed using the NJ method with the Kimura two-parameter option. Bootstrap analysis was conducted with 1000 replicates.

## Results

### Detection and isolation of waterfowl parvovirus

**Figure-1:**
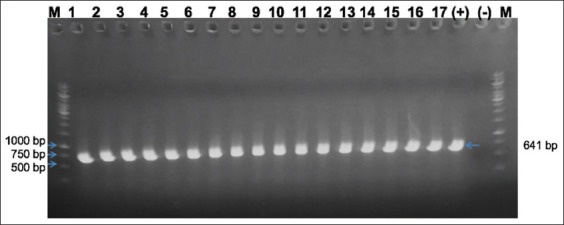
Detection of the waterfowl parvovirus genome in field samples. M=1kb DNA ladder; (+)=Positive control, (−)=Negative control, lanes 1–17=Collected samples from Han-GPV1901 to Han-GPV2104.

In total, 17 clinical samples were collected from duck farms infected with symptoms of SBDS during 2020–2022 (Table-1). The outbreaks showed typical clinical signs, including growth retardation, shortened beak, tongue protrusion, and weakened foot with morbidity of 15%–30%. A shortened beak was the most typical characterization and was observed at the age of 18–20 days, significantly affecting their ability to eat and drink. All samples were positive by PCR for the waterfowl parvovirus genome (Figure-1). Samples contaminated with other avian viruses, such as duck tembusu virus, duck enteritis virus, duck hepatitis virus Type 1 and Type 3, Newcastle disease virus, and influenza A virus, were also tested by PCR methods with the primers showed in Table-2 [13-19] to discard samples contaminated with other viruses. All of the samples were negative for these viral genomes.

**Figure-2:**
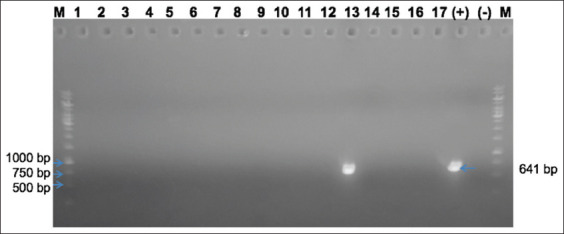
Identify of waterfowl parvovirus after 3^rd^ passage of embryonating duck eggs. M=1 kb DNA ladder, (+)=Positive control, (−)=Negative control; lanes 1–17=Collected samples from Han-GPV1901 to Han-GPV2104.

**Figure-3:**
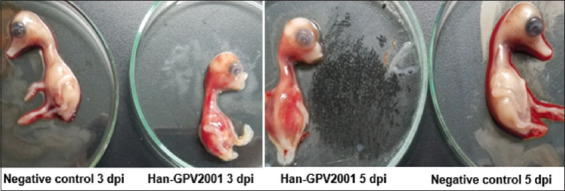
Han-GPV2001-infected duck embryos with spots of hemorrhage and beak abnormal. dpi=Day post-infection.

Viral isolation was performed by inoculating 10-day-old duck-embryonated eggs through the yolk sac route. The allantoic fluid and homogenates of the embryos’ internal organs, including liver, heart, and intestine, which were found to be negative with the hemolytic test using chicken blood, were collected and homogenated on day 6 post-inoculation and used to inoculate duck embryos for the next passage. After three passages, only Han-GPV2001 was positive for the waterfowl parvovirus by PCR (Figure-2). Han-GPV2001-infected duck embryos presented typical characteristics with abnormal beaks, hemorrhages, hematomas, and hemorrhagic internal organs (Figure-3).

### Determination of the virulence of the isolated virus

**Figure-4:**
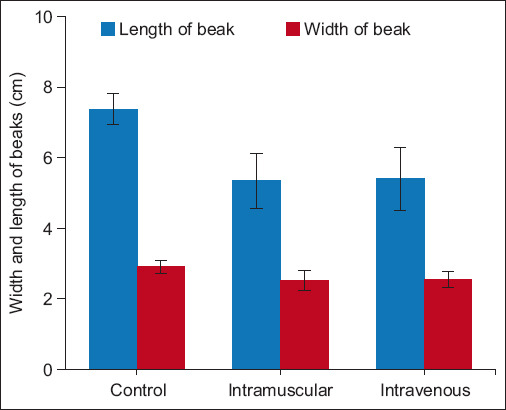
The width and the length of beaks of the infected duck with the Han-GPV2001 virus strain.

**Figure-5:**
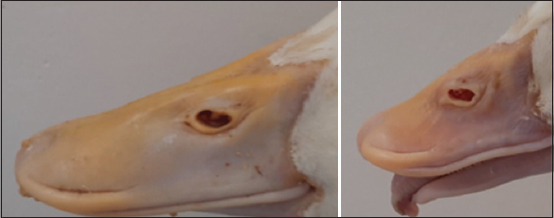
The beak of infected duck with Han-GPV2001 virus strain after 51-day post-infection (dpi). Dpi=Day post-infection.

The Han-GPV2001 virus was further inoculated into embryonated duck eggs through five passages using the yolk sac approach. Inoculated duck embryos died 72 h –120 h after inoculation, and the embryo mortality rate was 94.35% ± 0.96 6 days after infection. We tested the virulence of the isolated Han-GPV2001 strain at the sixth passage through the eggs. Three-day-old ducklings were intramuscularly and intravenously inoculated with the Han-GPV2001 virus strain at a dose of 10^4.03^ EID_50_/mL. The results indicated that 66.67%–80% of ducks infected with Han-GPV2001 showed clinical signs of SBDS (such as short beak, tongue protrusion, and growth retardation) and death depending on the method of inoculation, which were similar to those displayed in naturally infected ducks. The width and length of the beaks of the inoculated ducks differed significantly from those of the control ducks (Figure-4). The incidence of shortened beak, tongue protrusion, poor growth, fragile legs, low weight, and death was 80% in the intramuscular group and 66.67% in the intravenous group (Figure-5). On 21-day post-infection, the viral genome was detected in the blood and fecal samples from infected ducks.

### Genetic and phylogenetic analyses

It is possible to use entire genome sequencing, NS protein-coding gene sequencing, or VP1 structural protein coding gene sequencing [12, 22] to investigate the genetic relationship of the isolate with known waterfowl parvoviruses. The nucleotide identity of the partial NS and VP1 gene sequences was 99.83% and ranged from 99.24% to 99.39%, respectively, compared with the Han-GPV2001 and previous Vietnamese strains in GenBank. The Han-GPV2001 isolate shared the highest level of nucleotide identity (99.83%) with the GPV strains reported in China (GDQY1802, JS1, SDHZ1604/2016, and AH strains) when comparing the partial NS gene. Han-GPV2001 shared the highest nucleotide identity of 99.84% with the Chinese strains (HuN001 and GDQY1802 strains).

A phylogenetic tree based on partial NS gene sequences was constructed in this study to clarify the association of Han-GPV2001 with previously published virus strains. The tree shows that there are two genetic groups of waterfowl parvovirus, GPV, and MDPV. The classical GPV group was divided into two subgroups: The NGPV group [6, 12].

**Figure-6:**
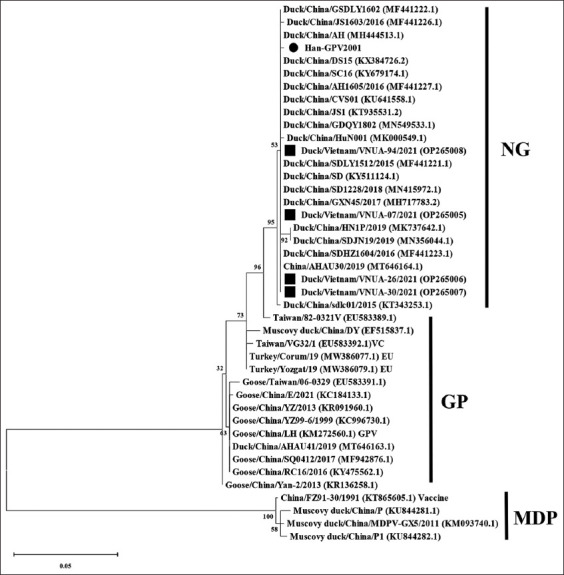
Phylogenetic tree was constructed based on the partial non-structural gene sequence of the Han-GPV2001 isolate, which was indicated in black circle. Previous Vietnamese waterfowl parvovirus strains were marked in black squares. The maximum likelihood method in the MEGA6 software was used to establish phylogenetic trees (1,000 bootstrap replicates). Numbers at each branch point indicate bootstrap values ≥50% in the bootstrap interior branch test.

**Figure-7:**
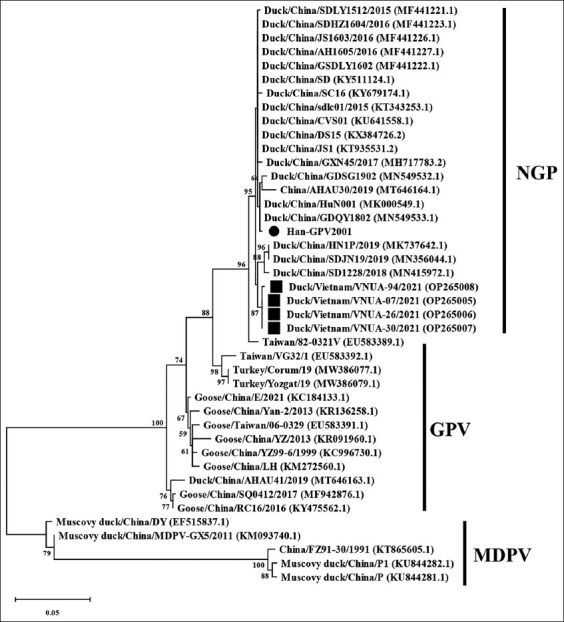
Phylogenetic tree was constructed based on the partial VP1 gene sequence of the Han-GPV2001 isolate, which was indicated in black circle. Previous Vietnamese waterfowl parvovirus strains were marked in black squares. The maximum likelihood method in the MEGA6 software was used to establish phylogenetic trees (1000 bootstrap replicates). Numbers at each branch point indicate bootstrap values ≥50% in the bootstrap interior branch test.

The phylogenetic analysis showed that the Han-GPV2001 isolate belongs to the NGPV group (Figure-6). The Han-GPV2001 virus strain was further classified based on the analysis of the VP1 gene sequence. The VP1-encoding nucleotide sequence obtained was compared with other accessible waterfowl parvovirus sequences available in GenBank. A comparison of the partial VP1 gene sequences showed that the Han-GPV2001 isolate had higher similarity with the NGPV group strains (Figure-7).

Nucleotide sequences and amino acid sequence comparisons revealed that only one nucleotide site in the Han-GPV2001 strain differed from that in the other strains (T instead of C), resulting in a difference in amino acid sequences from proline (P) to serine (S).

## Discussion

For the 1^st^ time, ducks infected with SBDS caused by NGPV in Vietnam were confirmed in 2019 [6]. In this study, GPV was isolated from the liver, heart, and spleen of duck flocks presenting clinical signs of SBDS with 15%–30% morbidity. After three blind passages in embryonated duck eggs, only one sample was successfully isolated as Han-GPV2001 from 17 samples collected. Phylogenetic analysis of the partial VP1 and NS gene sequences revealed that the Vietnamese isolate obtained in this study belonged to an NGPV strain that was closely related to previous Vietnamese and Chinese strains.

In this study, the Han-GPV2001 isolate resulted in 94.35% mortality after propagation in embryonated duck embryos at the sixth passage. Compared with previous research by Ning *et al*. [23], isolating virus from SBDS specimens onto embryonated goose eggs caused 57.14% embryo death/mortality on 10–14-day post-inoculation, and mortality probability reached 100% on two subsequent passages within 5–8-day post-inoculation. Clinical signals were observed in dead embryos at the beginning of the first passage [23]. The virulence of the isolated virus strain was higher than that reported after five passages on embryonated duck eggs, and the mortality rate with systemic hemorrhage was 60% at 72-h post-inoculation [24].

Infection with the Han-GPV2001 isolate in 3-day-old ducklings showed that SDBS was reproduced naturally with clinical signs similar to those of naturally infected ducks. Morbidity ranged from 66.67% to 80% depending on the type of infection. This result was similar to the morbidity rate observed with 70% infection and 5% mortality [2]. Meanwhile, the authors found that ducks showed the typical signs of SBDS accounting for only 20% of the group inoculated at 2 weeks of age, and no dead ducks were observed. There was no significant difference in the width of the beaks between the experimental and control groups, but the length of the affected ducks was shorter. However, when the infection was challenged at 2 weeks of age, there was no significant difference between them. The virulence of the Han-GPV2001 strain was higher than that of the previously reported strain, with 33.33% of ducks showing clinical signs of SBDS after infection [11]. The isolated strain had a lower virulence than the virus strain infected on 2-day-old geese [23], with 77.3%–93.35% mortality depending on the infected virus strain. All surviving infectious geese presented with growth retardation [23].

On the basis of the nucleotide sequences of the gene encoding NS and VP1, Han-GPV2001 was classified as an NGPV in this study.

## Conclusion

To the best of our knowledge, this is the first report of the successful isolation of GPV from ducks associated with SDBS in Vietnam. The Han-GPV2001 isolate was highly virulent when inoculated into 10-day-old duck embryos and 3-day-old ducklings. The mortality rate of duck embryos was 94.35% within 6 days after virus inoculation. Three-day-old ducks inoculated with 10^4.03^ EID_50_ of the virus stock through intramuscular and neck intravenous injection showed 80% and 66.67% clinical signs of SDBS, respectively. Phylogenetic analysis based on the partial NS and VP1 gene sequences revealed that the viral isolate obtained in this study belonged to NGPV and was closely related to previous Vietnamese and Chinese strains.

## Authors’ Contributions

NTH: Collected the samples in North Vietnam and did the virus isolation experiment. DVH and NTB: Prepared and conducted hemagglutination assay. TVK and NTBa: Performed and determined the virulence of the virus. CTNX and QTMH: Extracted DNA and conducted PCR. THT: Collected nucleotide sequencing and analysis. CTTH: Collected all data and drafted the manuscript. All authors have read, reviewed, and approved the final manuscript.

## References

[1] Chen H, Dou Y, Tang Y, Zheng X, Niu X, Yang J, Yu X, Diao Y (2016). Experimental reproduction of beak atrophy and dwarfism syndrome by infection in cherry valley ducklings with a novel goose parvovirus-related parvovirus. Vet. Microbiol.

[2] Palya V, Zolnai A, Benyeda Z, Kovacs E, Kardi V, Mato T (2009). Short beak and dwarfism syndrome of mule duck is caused by a distinct lineage of goose parvovirus. Avian Pathol.

[3] Chang P.C, Shien J.H, Wang M.S, Shieh H.K (2000). Phylogenetic analysis of parvoviruses isolated in Taiwan from ducks and geese. Avian Pathol.

[4] Wozniakowski G, Kozdrun W, Samorek-Salamonowicz E (2009). Genetic variance of Derzsy's disease strains isolated in Poland. J. Mol. Genet. Med.

[5] Chen S, Wang S, Cheng X, Xiao S, Zhu X, Lin F, Wu N, Wang J, Huang M, Zheng M, Chen S, Yu F (2016). Isolation and characterization of a distinct duck-origin goose parvovirus causing an outbreak of duckling short beak and dwarfism syndrome in China. Arch. Virol.

[6] Nguyen V.G, Dang H.A, Nguyen H.H, Nguyen T.B, Cao T.B.P, Huynh T.M.L (2019). The preliminary result on detection of waterfowl parvovirus in Hung Yen province 2019. Vietnam J. Agri. Sci.

[7] Dong H.V, Tran G.T.H, Nguyen H.T.T, Nguyen T.M, Trinh D.Q, Le V.P, Choowongkomon K, Rattanasrisomporn J (2022). Epidemiological analysis and genetic characterization of parvovirus in ducks in Northern Vietnam reveal evidence of recombination. Animals (Basel).

[8] Zadori Z, Erdei J, Nagy J, Kisary J (1994). Characteristics of the genome of goose parvovirus. Avian Pathol.

[9] Wang S, Cheng X.X, Chen S.Y, Lin F.Q, Chen S.L, Zhu X.L, Wang J.X, Huang M.Q, Zheng M (2016). Phylogenetic analysis of VP1 gene sequences of waterfowl parvoviruses from the Mainland of China revealed genetic diversity and recombination. Gene.

[10] Tsai H.J, Tseng C.H, Chang P.C, Mei K, Wang S.C (2004). Genetic variation of viral protein 1 genes of field strains of waterfowl parvoviruses and their attenuated derivatives. Avian Dis.

[11] Wan C.H, Chen H.M, Fu Q.L, Shi S.H, Fu G.H, Cheng L.F, Chen C.T, Huang Y, Hu K.H (2016). Development of a restriction length polymorphism combined with direct PCR technique to differentiate goose and Muscovy duck parvoviruses. J. Vet. Med. Sci.

[12] Shao H, Lv Y, Ye J, Qian K, Jin W, Qin A (2014). Isolation of a goose parvovirus from swan and its molecular characteristics. Acta. Virol.

[13] Wan C, Chen C, Cheng L, Liu R, Shi S, Fu G, Chen H, Fu Q, Huang Y (2019). Specific detection and differentiation of classic goose parvovirus and novel goose parvovirus by TaqMan real-time PCR assay, coupled with host specificity. BMC Vet. Res.

[14] Bian G, Ma H, Luo M, Gong F, Li B, Wang G, Mohiuddin M, Liao M, Yuan J (2019). Identification and genomic analysis of two novel duck-origin GPV-related parvovirus in China. BMC Vet. Res.

[15] Sun X, Liu E, Iqbal A, Wang T, Wang X, Haseeb A, Ahmed N, Yang P, Chen Q (2019). The dynamic distribution of duck Tembusu virus in the spleen of infected shelducks. BMC Vet. Res.

[16] Doan H. T, Le X. T, Do R. T, Hoang C. T, Nguyen K. T, Le T. H (2016). Molecular genotyping of duck hepatitis A viruses (DHAV) in Vietnam. J. Infect. Dev. Ctries.

[17] Boroomand Z, Jafari R.A, Mayahi M (2016). Molecular characterization and phylogenetic study of the fusion genes of Newcastle disease virus from the recent outbreaks in Ahvaz, Iran. Virusdisease.

[18] Xie Z, Pang Y.S, Liu J, Deng X, Tang X, Sun J, Khan M.I (2006). A multiplex RT-PCR for detection of type A influenza virus and differentiation of avian H5, H7, and H9 hemagglutinin subtypes. Mol. Cell Probes.

[19] Hansen W.R, Nashold S.W, Docherty D.E, Brown S.E, Knudson D.L (2000). Diagnosis of duck plague in waterfowl by polymerase chain reaction. Avian Dis.

[20] Wang J, Ling J, Wang Z, Huang Y, Zhu J, Zhu G (2017). Molecular characterization of a novel Muscovy duck parvovirus isolate:Evidence of recombination between classical MDPV and goose parvovirus strains. BMC Vet. Res.

[21] Hall T.A (1999). BioEdit:A user-friendly biological sequence alignment editor and analysis program for Windows 95/98/NT. Nucleic Acids Symp. Ser.

[22] Yu K, Ma X, Sheng Z, Qi L, Liu C, Wang D, Huang B, Li F, Song M (2016). Identification of goose-origin parvovirus as a cause of newly emerging beak atrophy and dwarfism syndrome in ducklings. J. Clin. Microbiol.

[23] Ning K, Liang T, Wang M, Dong Y, Qu S, Zhang D (2018). Pathogenicity of a variant goose parvovirus, from short beak and dwarfism syndrome of Pekin ducks, in goose embryos and goslings. Avian Pathol.

[24] Saleh A.A, Khoudeir M.H (2020). Preliminary studies on the virus causing outbreak of duckling short beak and dwarfism syndrome (SBDS) in Egypt. J. Appl. Vet. Sci.

